# Effect of the amount of organic trigger compounds, nitrogen and soil microbial biomass on the magnitude of priming of soil organic matter

**DOI:** 10.1371/journal.pone.0216730

**Published:** 2019-05-16

**Authors:** Domenico Paolo Di Lonardo, Wietse de Boer, Hans Zweers, Annemieke van der Wal

**Affiliations:** 1 Department of Microbial Ecology, Netherlands Institute of Ecology (NIOO-KNAW), Wageningen, The Netherlands; 2 Soil Biology Group, Wageningen University, Wageningen, The Netherlands; 3 National Institute for Public Health and the Environment, Bilthoven, The Netherlands; Sichuan Agricultural University, CHINA

## Abstract

Priming effects (PEs) are defined as short-term changes in the turnover of soil organic matter (SOM) caused by the addition of easily degradable organic compounds to the soil. PEs are ubiquitous but the direction (acceleration or retardation of SOM decomposition) and magnitude are not easy to predict. It has been suggested that the ratio between the amount of added PE-triggering substrate to the size of initial soil microbial biomass is an important factor influencing PEs. However, this is mainly based on comparison of different studies and not on direct experimentation. The aim of the current study is to examine the impact of glucose-to-microbial biomass ratios on PEs for three different ecosystems. We did this by adding three different amounts of ^13^C-glucose with or without addition of mineral N (NH_4_NO_3_) to soils collected from arable lands, grasslands and forests. The addition of ^13^C-glucose was equivalent to 15%, 50% and 200% of microbial biomass C. After one month of incubation, glucose had induced positive PEs for almost all the treatments, with differences in magnitude related to the soil origin and the amount of glucose added. For arable and forest soils, the primed C increased with increasing amount of glucose added, whereas for grassland soils this relationship was negative. We found positive correlations between glucose-derived C and primed C and the strength of these correlations was different among the three ecosystems considered. Generally, additions of mineral N next to glucose (C:N = 15:1) had little effect on the flux of substrate-derived C and primed C. Overall, our study does not support the hypothesis that the trigger-substrate to microbial biomass ratio can be an important predictor of PEs. Rather our results indicate that the amount of energy obtained from decomposing trigger substrates is an important factor for the magnitude of PEs.

## Introduction

Priming effects (PEs) are defined as short-term changes in the turnover of soil organic matter (SOM) caused by the input of easily degradable organic compounds (e.g. plant residues, root exudates, excretes of soil animals) to the soil [1]. So far, a reliable prediction of the direction (acceleration or retardation of SOM decomposition) and magnitude of PEs in response to organic carbon additions cannot be given. Several environmental factors influence PEs, such as the amount and chemical structure of added substrates [2–4], the inorganic nutrient availability [1,5] and the microbial biomass and community structure [6–8]. A meta-analysis [6] by Blagodatskaya and Kuzyakov (2008) indicated that the magnitude and direction of PEs are dependent on the ratio of the amount of added substrate to the size of the microbial biomass. Their analysis revealed a linear increase in PEs with increasing amount of trigger compounds as long as the added C substrate is less than 15% of the size of the microbial biomass. In contrast, an exponential decrease in PEs was found when the amount of trigger compounds was more than 50% of the size of the soil microbial biomass. This decrease in PEs with high amount of triggering compounds is suggested to be due to the so called preferential microbial substrate utilization [9,10] where soil microbes switch to utilize added easily degradable C sources instead of native soil C.

The meta-analysis of Blagodatskaya and Kuzyakov (2008) considered all publications on PEs with information on microbial biomass C. However, it is known that the structure and functioning of microbial communities and quality of soil organic matter greatly differ among ecosystems [11,12] and that this can have a strong impact on PEs [13]. Moreover, the studies taken into consideration in the meta-analysis included both single and multiple applications of the triggering substrates. Single and multiple applications will differently affect soil microbial biomass as well as community structure. These differences can have an impact on PEs [14–23].

The effects of concentrations of triggering compounds on PEs was recently tested [24]. In this study different amounts of the same trigger compound (glucose) were added to different soil ecosystems collected along an elevation gradient. The doses of added glucose were based on the initial size of the soil microbial biomass and they used multiple additions rather than a single one to resemble the temporal dynamics of labile C input in the field. The main finding was that PEs are increasing with higher amounts of trigger substrates and, therefore, not strongly related to the initial size of the soil microbial biomass. Yet, the magnitude of increase of PEs with increasing amount of glucose varied among the ecosystems included in the study.

The current study was already started when the paper of Liu et al. (2017) [24] appeared and had basically the same purpose: to investigate if the effects of trigger substrate to microbial biomass ratios on PEs are in agreement with the outcome of the meta-analysis of Blagodatskaya and Kuzyakov (2008). In particular, we were interested in this since most studies on PEs use an amount of trigger substrate that is at least 45% of soil microbial biomass [2,4,25,26], which is far higher than recommended by Blagodatskaya and Kuzyakov (2008). Based on the meta-analysis of Blagodatskaya and Kuzyakov (2008), we hypothesized that the trigger-substrate to microbial biomass ratio is an important predictor of PEs. To test the general validity of this hypothesis, we included three different ecosystems in our study, namely arable fields, grasslands and forests. Like most other studies, we have used single trigger substrate additions, whereas the study of Liu et al, (2017) used multiple applications.

Concurrently with addition of different amounts of a PE-triggering substrate (^13^C-glucose) we added ammonium nitrate (NH_4_NO_3_) to study the effect of nitrogen on PEs, as the availability of N can also influence the magnitude of PEs [5]. According to the “microbial nitrogen mining” hypothesis microbes use labile C as an energy source to decompose recalcitrant organic matter in order to obtain mineral N [27]. Hence, N addition may reduce mining for N and consequently SOM decomposition [28]. However, the N mining theory has been challenged as simultaneous addition of C and N was shown to stimulate rather than decrease priming [2,29,30]. Stimulation of decomposition can be driven by the stoichiometry of substrates, with the highest decomposition rates observed when the ratios of supplies of C and N to microbes match their demands [31,32]. Hence, simultaneous addition of C and N compounds can alleviate the stoichiometric constraint [28,33], causing a better match with N demands necessary for microbial growth and enzyme production and, consequently, resulting in higher PEs [2]. Based on these considerations and our previous results [2] we hypothesized that addition of N will not have a negative effect on PEs.

## Materials and methods

### Soil sampling and processing

In August 2015, soil (0–10 cm) was collected from three different ecosystems in the central part of the Netherlands [34], i.e. arable fields, beech forests and natural grasslands developed on abandoned arable fields. The arable fields were planted with maize (*Zea mays* L.), the dominant plant species in the forests was beech (*Fagus syslvatica* L.) and the natural grasslands were dominated by grasses such as common bent (*Agrostis capillaris* L.), tufted grass (*Holcus lanatus* L.) and forbs such as narrow-leave plantain (*Plantago lanceolata* L.) [34]. For each ecosystem type, we collected soils from four separate sites that were about 1 km apart, representing four ecosystem replicates [34]. In the laboratory, fresh soil from each plot was sieved (4 mm) and homogenized, removing fine roots and other plant debris. Field-moist soil was then stored at 4 °C until further use.

### Measurements of biotic and abiotic soil properties

Before the start of the experiments from each of our soil samples we collected a random subsample to determine biotic and abiotic soil conditions [34].

#### Soil chemical properties

Descriptions of the methods used for the evaluation of soil chemical properties are reported in [35]. Soil pH (soil: H_2_O,1:2 w:v), gravimetric moisture content (dried at 105°C, to constant mass) and maximum water holding capacity (WHC) was measured in fresh soil samples. Total C and N in all soil samples were measured by a combustion method using an elemental analyser (Thermo flash EA 1112, Thermo Fisher Scientific Inc.). Mineral N was extracted by shaking 10 g dry weight soil with 50 ml 1 M KCl for two hours. Concentration of N-NH_4_^+^ and N-NO_3_^-^ in the KCl extract were determined using an AutoAnalyzer (SEAL QuAAtro Segmented Flow Analysis system). The orthophosphate fraction from the soils was extracted in a 1:20 (w/v) ratio with a 0.5 molar solution of NaHCO_3_ at pH 8.5. Concentration of P-(PO_4_) in the extracts was determined by an AutoAnalyzer (SEAL QuAAtro Segmented Flow Analysis system). Chemical properties of the soils are listed in Table 1.

**Table 1:** Mean values (±SE) of chemical soil properties for each of the three soil types (arable, grassland, forest).

Soil	pH	C:N ratio	N-NO_3_ [mg kg^-1^ dw soil]	N-(NH_4_) [mg kg^-1^ dw soil]	P-(PO_4_) [mg kg^-1^ dw soil]
**Arable**	5.7 ± 0.3	17.4 ± 0.4	31.5 ± 5.4	0.2 ± 0.1	231.5 ± 15.9
**Grassland**	5.5 ± 0.1	17.7 ± 0.9	5.1 ± 0.5	0.3 ± 0.3	99.7 ± 6.8
**Forest**	3.9 ± 0.3	24.1 ± 0.4	2.3 ± 0.4	1.6 ± 0.1	18.6 ± 1.8

#### DNA extractions and quantitative PCRs

To determine bacterial biomass we extracted DNA from soils using the PowerSoil DNA Isolation Kit (MOBIO Laboratories, Carlsbad, California, USA) according to the manufacturer’s instruction with some modifications: after adding solution C1 (causing cell lysis), samples were incubated at 60 °C for 30 min; after adding solution C6 (releasing DNA from spin filter), samples were incubated at 30 °C for 10 min. Total DNA was quantified using a NanoDrop ND-1000 Spectrophotometer (Bio-Rad Laboratories Inc.).

Briefly, each qPCR reaction for bacterial quantification (total volume 15 μl) consisted of 7.5 μl of Sybergreen (iTaq Universal SYBR Green Supermix), 0.6 μl of forward primer (Eub 338, 10 pmol μl^-1^) [36], 0.6 μl of reverse primer (Eub 518, 10 pmol μl^-1^) [36], 0.6 μl of reverse primer (Eub 518, 10 pmol μl^-1^)[37], 3.3 μl Nucleic acid free water (Sigma) and 3 μl of DNA. Plasmid Ter331 (Collimonas 16S) was used as a standard for the quantification. The PCR program consisted of an initial denaturation step at 95 °C for 2 min, followed by 40 cycles of 95 °C for 10 sec, 53 °C for 10 sec and 72 °C for 25 sec. The qPCRs were performed with a Rotor-Gene RG-3000 (Corbett research). For each template DNA we analysed four biological replicates in duplicate. The qPCR results, expressed as 16S rRNA gene copy numbers g^-1^ of dry weight soil, were used to calculate the bacterial cell numbers using a conversion factor of 4.1 copies per cell [38] and bacterial biomass C as described by [39].

#### Fungal biomass

Ergosterol, a sterol found in fungal membranes, was used as a biomarker for fungal biomass. We used the protocol described by [40]. Briefly, 4 g of moist soil was shaken with 6 ml of methanol in the presence of glass beads, to disrupt the fungal mycelium and to release the ergosterol into the extractant. After centrifugation and filtration, ergosterol was measured on a 1260 Bio-inert LC coupled with a 6460 QQQ (Agilent, Santa Clara, USA). The results obtained, expressed as mg kg^-1^, were used to calculate the fungal biomass using a conversion factor of 5.4 Conversion factors of 5.4 mg ergosterol g^-1^ biomass C [41].

### Mesocosm set-up

Glass bottles (500 ml) were filled with moist soil equivalent to 80 g of dry weight and the soil was pre-incubated in a climate chamber for an acclimatization period of 15 days at 20°C. The acclimatization period was based on the results of a previous pilot experiment (data not shown), where soil CO_2_ efflux rates were regularly checked to confirm stabilization of the soil microbial activity.

Soil samples were mixed with single additions of three different aqueous solutions of uniformly labelled 99 atom% ^13^C-glucose (Campro Scientific GmbH). The amount of glucose-C added was equivalent to 15%, 50%, and 200%, of the microbial biomass carbon (Table 2) [6]. Besides the addition of the three different glucose solutions, half of the mesocosms also received an aqueous solution of NH_4_NO_3_ to establish a final C-glucose to N ratio of 15:1 [8]. Controls consisted of soils without addition. The solutions were stirred into the soils to ensure a homogeneous mixture. Each treatment included four soil replicates per ecosystem type. Soils were incubated at 20 °C in the dark for 30 days. The soil moisture was maintained at 60% of the water holding capacity throughout the incubation period by weighing the microcosms once a week and watering with deionized water when needed. After 4 days (time period based on the development of total CO_2_ respiration) and 30 days (end of the experiment) of incubation, soil was sampled from each treatment using an ethanol- cleaned tweezer to minimize contamination. These soil samples were used for microbial biomass measurements. The samples were frozen (-20 °C) prior to DNA and ergosterol extractions.

**Table 2:** Mean values (±SE) of initial fungal and bacterial biomass (mg C g^-1^ dw soil) of the sampled soils and amount of ^13^C-glucose added (mg C g^-1^ dw soil) to the soils in a quantity of C equal to 15%, 50%, and 200% of the microbial biomass carbon.

	Fungal biomass	Bacterial biomass	Tot Microbial biomass	^13^C-glucose (mg C g^-1^ dw soil)		
Soil type	(mg C g^-1^ dw soil)			15%	50%	200%
Arable	0.19 ± 0.01 ^a^	0.21 ± 0.04 ^a^	0.40 ± 0.05 ^a^	0.06 ± 0.01	0.20 ± 0.03	0.81 ± 0.10
Forest	0.52 ± 0.04 ^b^	0.17 ± 0.04 ^a^	0.69 ± 0.05 ^c^	0.10 ± 0.10	0.34 ± 0.03	1.38 ± 0.11
Grassland	0.28 ± 0.03 ^a^	0.30 ± 0.02 ^a^	0.59 ± 0.05 ^ab^	0.09 ± 0.09	0.29 ± 0.03	1.17 ± 0.10

Statistically significant differences (P < 0.05) are marked with different letters. Fungal and bacterial biomass are estimated on basis of ergosterol (fungal) and qPCR-16SrDNA (bacteria).

### CO_2_ measurements

For CO_2_ efflux measurements, the bottles containing the soils were tightly closed 24 hours before sampling. Headspace CO_2_ was sampled through the lid septa and directly injected into 5.9 mL evacuated Exetainer vials (Labco Ltd., Buckinghamshire, UK). We sampled at 0.2 (5 hours), 1, 2, 3, 4, 5, 7, 9, 11, 15, 21, 30 days of incubation (12 sampling times in total). An aliquot of the gas samples (250 μl for each vial) was injected using an auto-sampler in the Ultra GC gas chromatograph (Interscience, Breda, The Netherlands) equipped with a flame ionization detector (FID) and a Rt-QBOND (30 m, 0.32 mm, ID) capillary column. Helium was used as carrier gas and the oven temperature was kept at 50 °C with a flow of 5 ml. For the analysis of the ^13^C-CO_2_ we used a Thermo Scientific gaschromatograph with combustion interface (Conflo III) system connected to a Thermo Scientific Delta V Plus isotope ratio mass spectrometer (Thermo Scientific, Bremen, Germany). A second aliquot of the gas samples (250 μl) was injected into the split injector (split ratio 1:10) and eluted with helium (5 ml/min) on Rt-QBOND (30 m, 0.32 mm, ID) capillary column at 31°C. The reference gas was calibrated with Methane δC (VPDB) -38.25 (Arndt Schimmelmann, Indiana University, Bloomington, USA). CO_2_ concentrations of 12 air samples per bottle were used to calculate the cumulative respiration for the whole incubation period. Cumulative fluxes were calculated by linear interpolation between measuring times.

### Priming effect calculations

The percentage of respired CO_2_ derived from ^13^C substrate was calculated for all treatments and sampling times according to the formula:

%Csubstratederived=[(δC-δT)/(δC-δL)]×100 (1)

Where δC is the δ^13^C value of the respired CO_2_ from control soils, δT is the δ^13^C value in respired CO_2_ from treated soils and δL is the δ^13^C value of the labelled substrate [42]. Data were expressed in μg C g^−1^ dry weight soil. PE was then calculated as the total respired CO_2_ in treated soils minus the substrate-derived CO_2_ and minus the respired CO_2_ in the control and expressed in μg C g^−1^ dry weight soil [13]:

PrimedC=Ctotal–Csubstrate–Ccontrol (2)

### Statistical analyses

To test how the amount of added glucose, nitrogen addition and their interactions affected glucose-derived CO_2_, PEs and microbial biomass (measured at fourth and thirtieth day of incubation) per soil type, we used two-way ANOVA. Data grouped per soil type with or without N addition were analysed with a one-way ANOVA, followed by post-hoc Tukey’s test, to determine differences between glucose additions. In case of unequal variances among treatments, statistical comparisons were performed by Tamhane’s test. We used one-sample T-tests to test whether PEs were different from zero. We used a regression analysis to test the relationship between the amount of added ^13^C-glucose (15%, 50%, and 200% of the microbial biomass C) and PEs. We used Pearson’s correlation coefficient to test for the relationships between substrate derived C and total respired C, and between substrate derived C and primed C. Statistical analyses were performed using IBM SPSS Statistics 22.

## Results

### CO_2_ effluxes and priming effect

The highest CO_2_ effluxes were observed in the arable and forest soils that received the highest amount of glucose (C-glucose equal to 200% of the microbial biomass carbon) (P < 0.05). In contrast, the lowest levels of CO_2_ evolution were seen for grassland soils amended with the highest amount of glucose (S1 Fig). Almost none of the additions of C-glucose equal to 15% and 50% of the microbial biomass carbon resulted in significant extra total CO_2_ evolution as compared to the control (P > 0.05). For grassland soils there were no significant differences in CO_2_ evolution for any of the carbon and nitrogen additions (S1 Fig) (P > 0.05).

**Fig 1:**
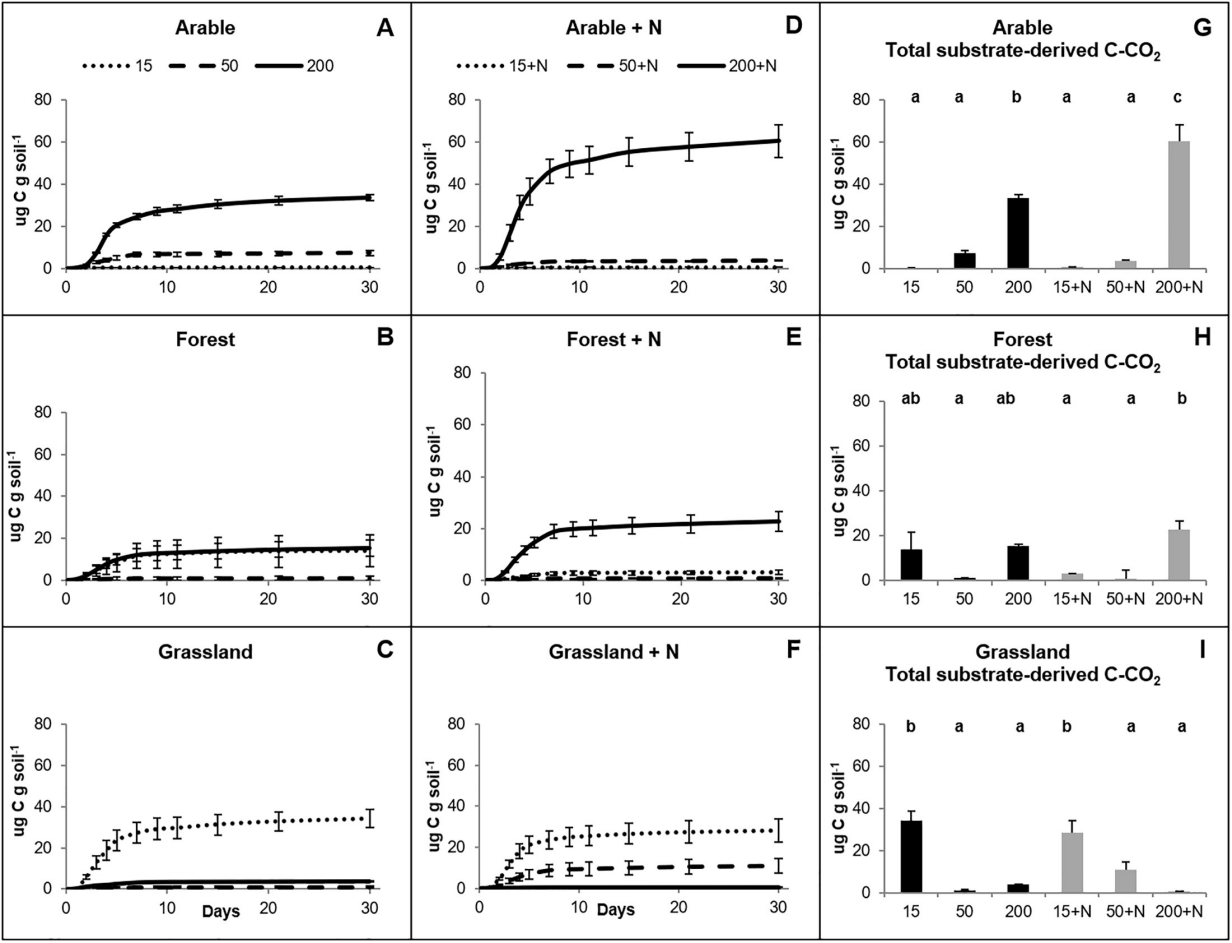
Substrate-derived CO_2_ (μg C-CO_2_ g dw soil^-1^) in soils from three ecosystems as induced by three different amounts of ^13^C-glucose (15%, 50%, and 200% of the microbial biomass carbon). A-F: Cumulative accumulation of substrate-derived CO_2_ over 30 days of incubation. G-H: Total substrate-derived CO_2_ after 30 days of incubation. N: NH_4_NO_3_. Statistically significant differences (P < 0.05) are marked with different letters. NS: no significant differences. Error bars represent standard errors (n = 4). Two-way ANOVA results are reported in S1 Table.

The CO_2_ evolution pattern was generally the same as compared to the release of substrate-derived C (^13^CO_2_, released from labeled glucose) (Fig 1). The positive relationship between substrate-derived CO_2_ and total soil respiration is confirmed by correlation analysis (R^2^ = 0.4577; P < 0.001; S2A Fig). Yet, the strength of the relationship between substrate derived CO_2_ and total respiration decreased for the different soils following this order: arable soils (R^2^ = 0.6977; P < 0.0001) > grassland soils (R^2^ = 0.3576; P = 0.002) > forest soils (R^2^ = 0.2938; P = 0.006). The highest amounts of substrate-derived C were observed for arable and forest soils that received C-glucose equal to 200% (P < 0.05; Fig 1G and 1H). On the contrary, for the grasslands soils the amount of substrate-derived C were highest in the treatments with the lowest amount of added glucose (P < 0.05; Fig 1I).

**Fig 2:**
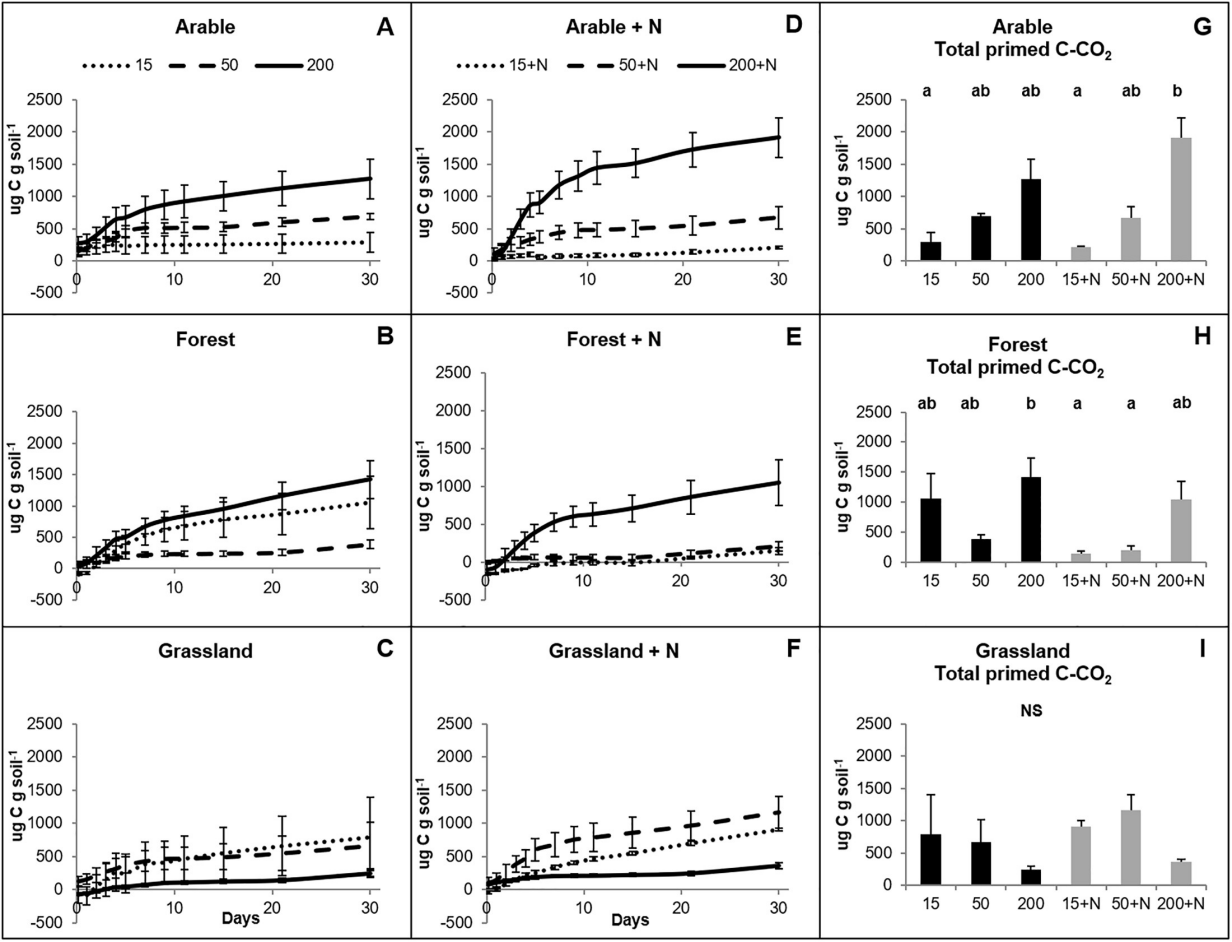
Primed CO_2_ (μg C-CO_2_ g dw soil^-1^) in soils from three ecosystems as induced by three different amounts of ^13^C-glucose (15%, 50%, and 200% of the microbial biomass carbon) after 30 days of incubation. A-F: Cumulative accumulation of primed CO_2_ over 30 days of incubation. G-H: Total primed CO_2_ after 30 days of incubation. N: NH_4_NO_3_. Statistically significant differences (P < 0.05) are marked with different letters. NS: no significant differences. Error bars represent standard errors (n = 4). Two-way ANOVA results are reported in S1 Table.

After 30 days of incubation, glucose had induced a positive PE (increase of respiration of unlabeled C) for almost all the soils (Fig 2; S2 Table). Primed C in arable soils increased with increasing amount of glucose added (Fig 2G) (P < 0.05). In forest soils this pattern was less clear although the highest amounts of primed C were also seen for the highest glucose additions (Fig 2H). The responses of the grassland soils to the different amount of glucose added were not significantly different in magnitude from each other in all treatments (P > 0.05). However, PE becomes significantly different from zero when glucose additions were combined with mineral N additions (S1 Table). For several additions in the forest and grassland soils we observed an initial (< 10 days) negative PE (Fig 2).

**Fig 3:**
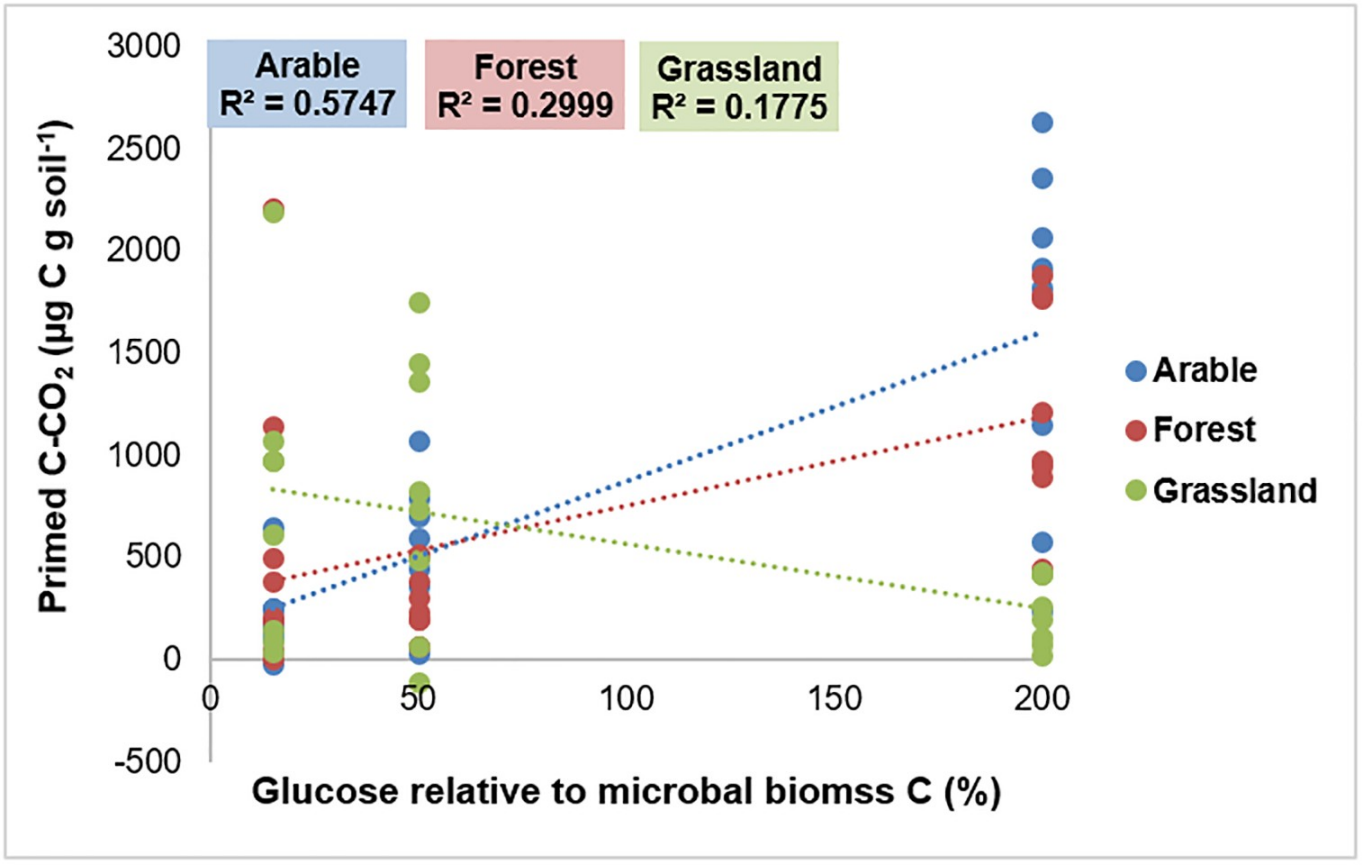
Regression analysis for the amount of added C-glucose expressed as % of microbial biomass (15%, 50% and 200%) versus primed C-CO_2_. Treatments are grouped together according to the ecosystems from which the soils were obtained.

The regression analysis showed a significant positive linear relationship between the amount of the added C expressed as % of the microbial biomass C and primed C for arable soils (R^2^ = 0.5747; P < 0.0001) and forest soils (R^2^ = 0.2999; P = 0.006), respectively (Fig 3). For the grassland soils this was a negative relationship (R^2^ = 0.1775; P = 0.04).

We found a positive correlation between substrate-derived CO_2_ and PEs for the different ecosystems (S2B Fig). In this case the strength of the positive relationship between substrate derived CO_2_ and PEs decreased from the arable soils (R^2^ = 0.72; P < 0.0001) to forest soils (R^2^ = 0.2702; P = 0.009) and grassland soils (R^2^ = 0.1856; P = 0.03).

The ratio between primed C to substrate-derived C was highest for the arable soils amended with the lowest amount of C-glucose combined with N (P < 0.05; S3 Fig). This relative strong impact of low doses of glucose on PEs was not seen for the soils originating from natural ecosystems (S3B and S3C Fig).

In general, addition of N had no significant effect on glucose-derived C and PEs (P = 0.347 and 0.581, respectively; S1 Table). It stimulated higher substrate derived respiration only in arable soils amended with C-glucose equal to 200% of the microbial biomass carbon (P < 0.01) and had the same tendency for primed C (P = 0.007) (Figs 1G and 2G).

### Microbial biomass

**Fig 4:**
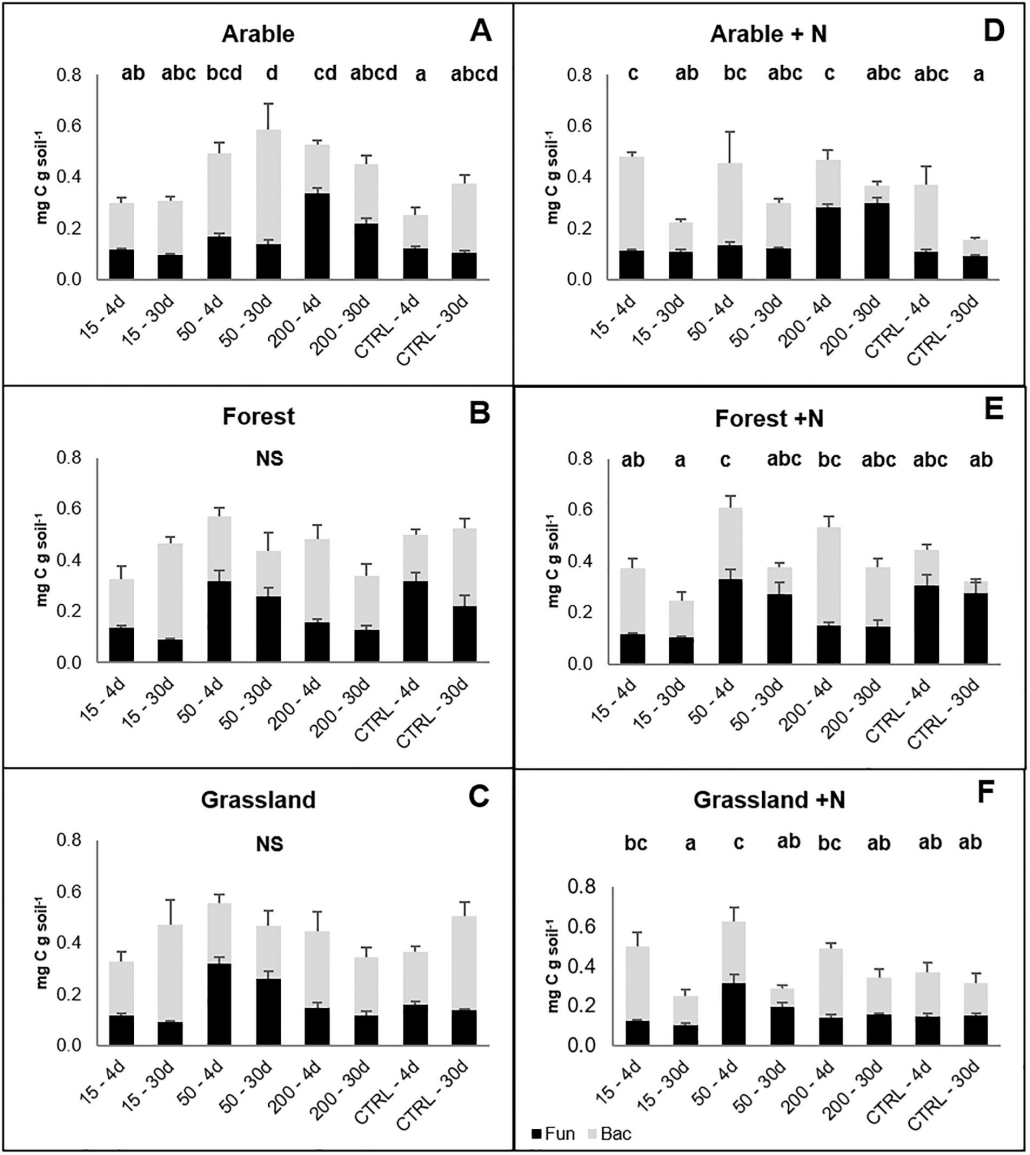
Microbial biomass (fungi + bacteria, mg C g dw soil^-1^) measured at 4 and 30 days of incubation of glucose-amended soils obtained from three ecosystems. Fungal and bacterial biomass are estimated on basis of ergosterol (fungal) and qPCR-16SrDNA (bacteria). 15, 50 and 200 indicate the quantity of glucose-C added representing 15%, 50%, and 200% of the initial microbial biomass carbon. 4d: fourth day of incubation. 30d: thirtieth day of incubation. CTRL: control treatment. N: NH_4_NO_3_. Statistically significant differences (P < 0.05) are marked with different letters. NS: no significant differences. Error bars represent standard errors (n = 4). Black columns: fungal biomass. Grey columns: bacterial biomass. Two-way ANOVA results are reported in S3 Table.

Initial microbial biomass was highest for forest soils, followed by grassland and arable soils (P < 0.05, Table 2). In addition, we found the highest initial fungal biomass in forest soils (P < 0.05), whereas there were no differences between arable and grassland soils. The three ecosystems did not differ in terms of soil bacterial biomass (P > 0.05, Table 2). After 4 days and 30 days of incubation, soils were sampled from each treatment to estimate the microbial biomass carbon. Generally, the microbial biomass remained constant throughout the incubation period, showing no particular differences (P < 0.05) among arable soils and no significant differences among forest and grassland treatments (Fig 4, S3 Table). The proportion of fungal and bacterial biomass fluctuated between treatments but without a consistent pattern. Yet, we observed a trend for all the treatments amended with nitrogen, namely a decrease in total microbial biomass over time (Fig 4D, 4E and 4F). This appeared to be mainly caused by a decrease in bacterial biomass (S4 Fig).

## Discussion

### Amount of trigger compound and priming effects

Linear regression between the amount of added C (expressed % of the initial microbial biomass) and primed C revealed contrasting results for the different ecosystems. The meta-analysis by Blagodatskaya and Kuzyakov (2008) has indicated that additions of trigger compounds up to 15% of microbial biomass C induce a linear increase in PEs. In contrast, when the added amount of trigger compounds is higher than 50% of the microbial biomass C, an exponential decrease in PEs was seen. Our study could not confirm their findings and therefore the ratio between the amount of added C to the size of the soil microbial biomass does not appear to be a universal predictor for soil organic matter PEs. A similar conclusion has recently been drawn [24].

In contrast to our observations, Liu and colleagues (2017) found a positive linear relationship between increasing C additions and PEs in the natural ecosystems they studied, including grassland and forest ecosystems. These contrasting results might be related to different composition and structure of the soil microbial communities investigated in the two studies [43]. In our case, the strongest positive relationship of PEs with the amount of added glucose was seen for arable soils. A common agricultural practice is to amend soils with different organic matter residues such as manure and compost. Part of these materials are easily degradable as indicated by initial high respiration rates after addition to arable soils. Therefore, microbes in agricultural soils may be better adapted to receive high inputs of easily degradable organic matter and this could be the reason why they can cope better with temporary high organic carbon additions than soil microbes in natural ecosystems [44–46].

We found a positive correlation between substrate-derived C (^13^C) and primed C (^12^C) albeit that the strength of this correlation was different for the three ecosystems included. Arable soils showed the strongest correlation, followed by forest and grassland soils. These results indicate that the amount of energy obtained from decomposing trigger substrates is an important factor that defines the magnitude of PEs [47]. A strong positive correlation between substrate derived CO_2_ and primed CO_2_ was recently also observed [25]. Yet, the accumulation curves between substrate-derived C and primed C differed (Figs 1 and 2). Substrate-derived C accumulation was completed during the first week of incubation whereas primed C increased until the end of the experiment (30 days). This has been reported before and indicates that the initial microbial activation by the added glucose continued for a longer period [6,48].

Arable soils showed a different pattern of PE stimulation than natural soils as the lowest amount of glucose resulted in the highest ratio between primed C to substrate-derived C, in particular when glucose was combined with N (S3 Fig). This indicates a relatively stronger impact of low trigger substrate concentrations on PEs, which may be ascribed to the higher temporal heterogeneity of availability of energy sources in arable soils due to agricultural management practices such as fallow followed by addition of organic fertilizers. In such managed arable soils, microbes may have a strategy to become highly activated when easily degradable substrates become available [49,50].

It is largely assumed that lack of N in soils will induce high PEs since soil microbes are triggered to mine SOM to acquire this nutrient [5,24]. Following this so-called microbial N mining theory, a less strong PE is expected when C substrate is added together with N. In line with our hypothesis, we found that N addition had no or little effect on PEs. Arable and grassland soils did not differ in C:N ratio, while forest soils had the highest one. Hence, according to the N mining theory, the strongest negative effect of N addition on PEs could have been expected for the forest soils. This was not the case, indicating that other SOM properties are probably more important for describing the effect of N on PEs [51]. Several other studies did also show that simultaneous addition of C and N can increase or have no effect on PEs [2,29,30,34]. In line with these studies N mining theory was recently challenged [25] since in its current form it does not contribute to an explanation for PEs.

In grassland and forest soils, amended with the lowest glucose input, we observed a temporary decrease in the decomposition of SOM after the first few days of incubation. Negative PE was previously shown in the early phases after C addition to soils [2,24,52]. The shift of microbes from SOM decomposition to uptake of added C and N substrates is indicated to be the underlying mechanism of negative PEs [9,48]. Liu et al. (2017) found an overall negative PE for the whole incubation period with multiple low C additions and attributed this to a minimum amount of energy (threshold) needed to overcome N limitation. Qiao et al. (2016) indicated that different mechanisms can be responsible for negative PEs depending on the intrinsic C:N ratios of soil organic matter and C:N ratios of the trigger substrates. In our case, negative PE is followed by positive PE and is therefore more likely to be the result of initial activation of microbes using internal reserve material (negative apparent PE) [6].

The lack of respiration response (S1 Fig) and the very low PEs that we have seen in the grassland soils that received high C substrate additions might be due to a glucose oversaturation of microorganisms. Microbes present in the grassland samples receiving the highest amount of ^13^C-glucose started using this substrate but due to possible osmotic stress the total activity subsequently declined [53].

The discrepancy in the results between our work and other studies investigating PEs might be due to the differences we had in the experimental approaches, such as the ecosystems under investigation, the length of the experiments, the simultaneous amendments of C and N *versus* only C additions and the frequency of C input (single *versus* repeated C additions). With respect to the latter, single *versus* multiple additions might influence PE differently [13] as they have different impacts on the ability of microbes to invest energy in the synthesis of SOM-degrading enzymes [7,24,25]. Single substrate applications represent short-term pulses of easily accessible and degradable substrates that produce hotspots of microbial activity that induce accelerated decomposition process rates [54].

### Microbial biomass response

In our study the microbial biomass remained overall constant during the whole incubation period of the experiment although we found differences in PEs among soils. Generally, glucose did not stimulate the growth of fungi and bacteria differently, and both microbial groups appeared to play a role in PEs. The addition of the trigger compound could have served as energy source for the soil microbial community, stimulating the production of extracellular enzymes with subsequent increase in the decomposition of SOM [55].

We observed a decrease in microbial biomass in all treatments amended with N. Reduction of bacterial and fungal biomass in response to N fertilization is consistent with previous studies [56–58]. The addition of glucose and N to the soil mesocosms might have altered the demands necessary for microbial growth [59]. Furthermore, the effects of N fertilization can depend on the soil conditions prior to fertilization [56].

## Conclusions and perspectives

In this study we investigated the effect of different ratios of the amount of organic trigger compounds to initial soil microbial biomass on PEs. We did not find the contrasting effects of low and high ratios as predicted by a previous meta-analysis [6]. Our results support those of Liu et al. (2017) with respect to the lack of predictability of trigger substrate to microbial biomass ratios but only partly with respect to a consistent increase of positive PEs with increasing concentrations of trigger substrates. In arable and forest soils the primed C increased with an increasing amount of added glucose, but this was not the case for grassland soils. Hence, the proposed predictive value of trigger-substrate concentrations for explaining PEs [24] requires more investigations. Yet, we observed a significant relationship between the mineralization of the added trigger substrate and PEs for all soil types, indicating the importance of energy obtained from trigger-substrates for PEs. N additions together with glucose had little or no effect on PEs, rejecting the N mining theory. To be able to explain mechanisms of PEs and integrate PEs in global soil carbon models, more studies of soils with different properties are needed. Furthermore, it is important to understand how the quality and quantity of added compounds affect PEs in arable soils in order to improve the management of soil organic carbon dynamics.

## Supporting information

S1 Fig Total CO_2_ (μg C-CO_2_ g dw soil^-1^) in soils from three ecosystems as induced by three different amounts of ^13^C-glucose (15%, 50%, and 200% of the microbial biomass carbon) after 30 days of incubation. A-F: Cumulative accumulation of total CO_2_ over 30 days of incubation. G-H: total CO_2_ after 30 days of incubation. CTRL: control treatment. Statistically significant differences (P < 0.05) are marked with different letters. NS: no significant differences. N: NH_4_NO_3_. Error bars represent standard errors (n = 4).
(TIF)

S2 Fig Pearson correlation analysis for substrate-derived C-CO_2_ versus total C-CO_2_ (A) and for substrate-derived C-CO_2_ versus primed C-CO_2_ (B). Treatments are grouped together according to the soil type.
(TIF)

S3 Fig Ratio between primed CO_2_ and substrate-derived CO_2_ after 30 days of incubation of glucose-amended soils from three ecosystems. N: NH_4_NO_3_. Statistically significant differences (P < 0.05) are marked with different letters. NS: no significant differences. Error bars represent standard errors (n = 4).
(TIF)

S4 Fig Fungal and bacterial biomass (mg C g^-1^ dw soil ± SE) estimated on basis of ergosterol (fungal) and qPCR-16SrDNA (bacteria) measurements at 4 and 30 days of incubation. 15, 50 and 200 represent the quantity of C added as equal to 15%, 50%, and 200% of the initial microbial biomass carbon. 4d: fourth day of incubation. 30d: thirtieth day of incubation. CTRL: control treatment. N: NH_4_NO_3_.
(TIF)

S1 Table Two-way ANOVA results of the effect of amount of glucose additions (15%, 50%, and 200% of the microbial biomass carbon), nitrogen addition (yes/no) and their interactions on the amount of glucose-derived C (μg C-CO_2_ g soil^-1^), primed C (μg C-CO_2_ g soil^-1^). df represents the numerator, denominator degrees of freedom. (DOCX)

S2 Table One-sample T test (test value = 0) on significance of primed C-CO_2_ accumulation. 15, 50 and 200 represent the quantity of C added as equal to 15%, 50%, and 200% of the microbial biomass carbon. N: NH_4_NO_3_. *: Significant values (P < 0.05).
(DOCX)

S3 Table Two-way ANOVA results of the effect of amount of glucose additions (15%, 50%, and 200% of the microbial biomass carbon), nitrogen addition (yes/no) and their interactions on the soil microbial biomass measured at 4^th^ and 30^th^ days of incubation. df represents the numerator, denominator degrees of freedom. (DOCX)
