# Supplementary figures and images for: Effect of the amount of organic trigger compounds, nitrogen and soil microbial biomass on the magnitude of priming of soil organic matter
Source: PLoS One. 2019 May 16;14(5):e0216730. doi: 10.1371/journal.pone.0216730 (PMC6522013; doi:10.1371/journal.pone.0216730)

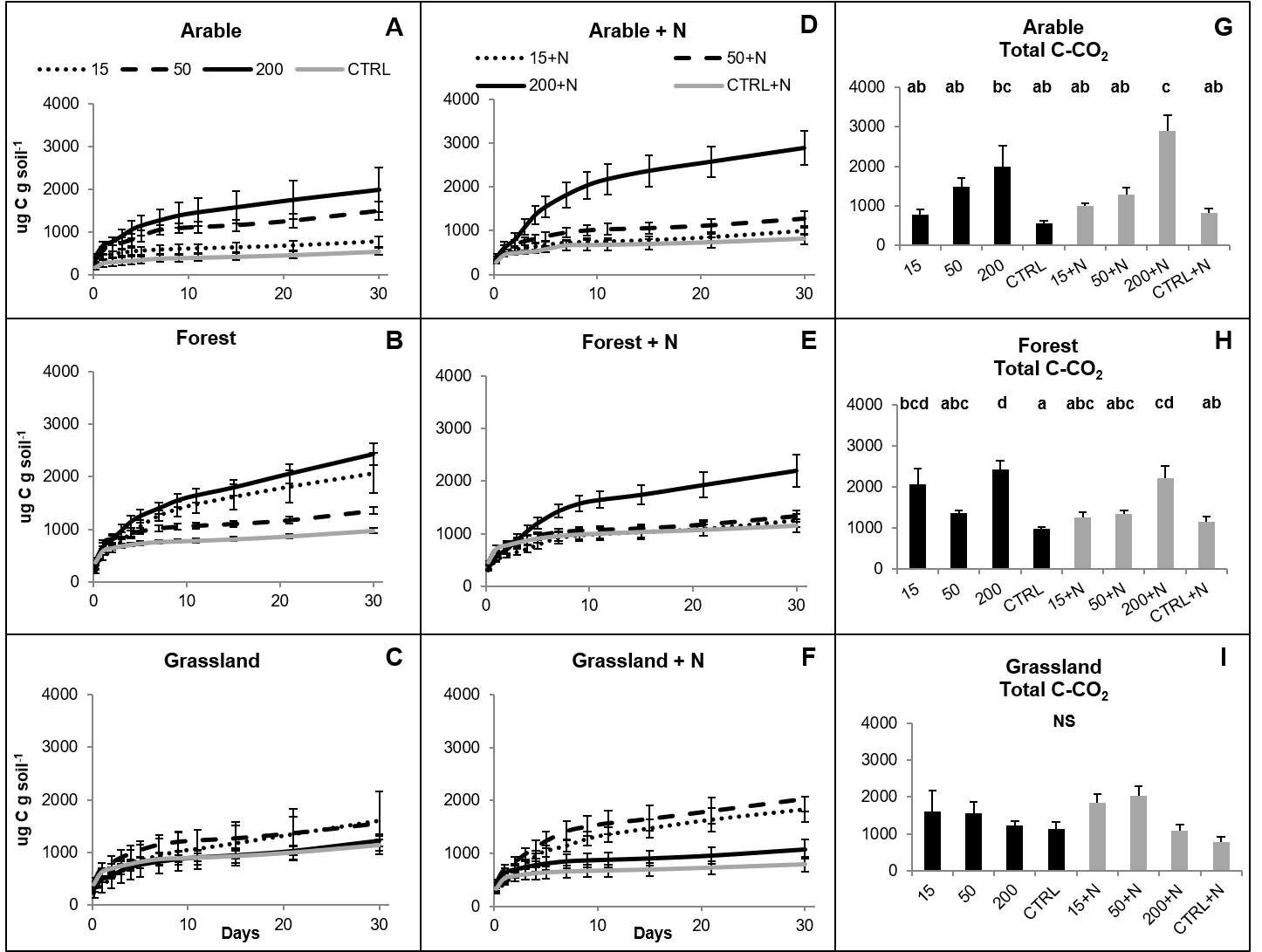

Supplement: S1 Fig — A-F: Cumulative accumulation of total CO2 over 30 days of incubation. G-H: total CO2 after 30 days of incubation. CTRL: control treatment. Statistically significant differences (P < 0.05) are marked with different letters. NS: no significant differences. N: NH4NO3. Error bars represent standard errors (n = 4). (TIF) [file pone.0216730.s001.tif]

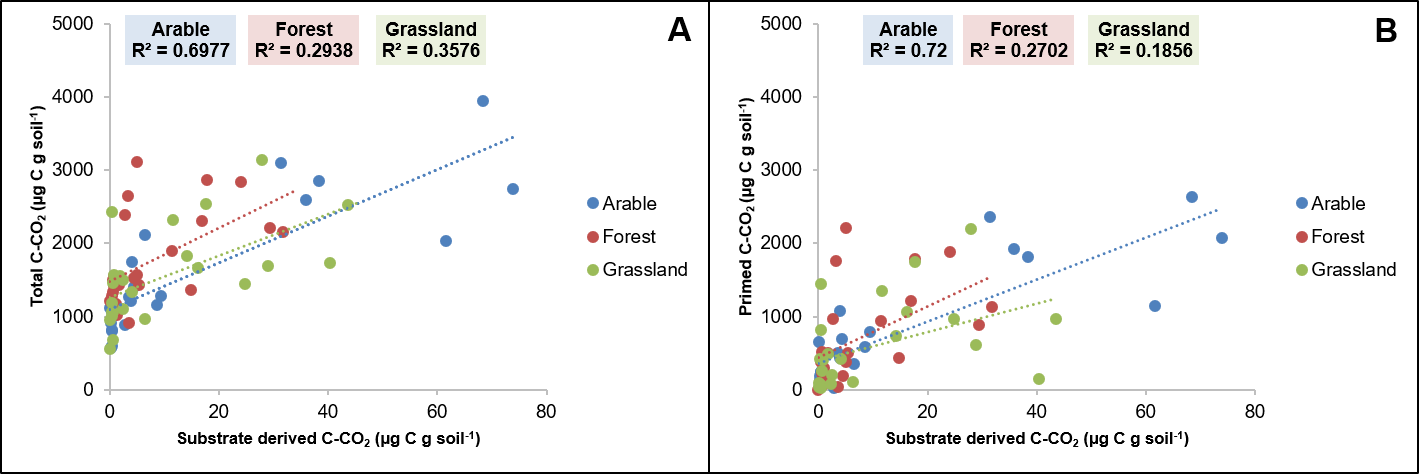

Supplement: S2 Fig — Treatments are grouped together according to the soil type. (TIF) [file pone.0216730.s002.tif]

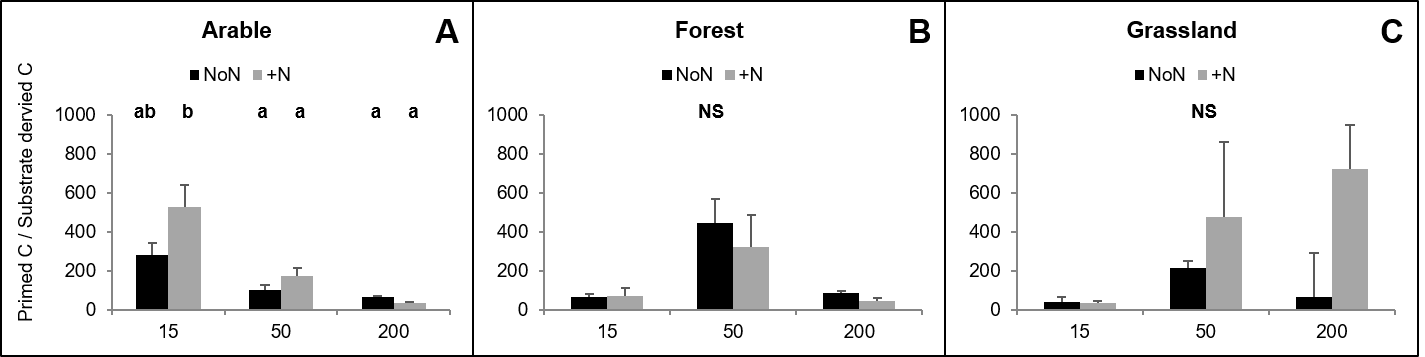

Supplement: S3 Fig — N: NH4NO3. Statistically significant differences (P < 0.05) are marked with different letters. NS: no significant differences. Error bars represent standard errors (n = 4). (TIF) [file pone.0216730.s003.tif]

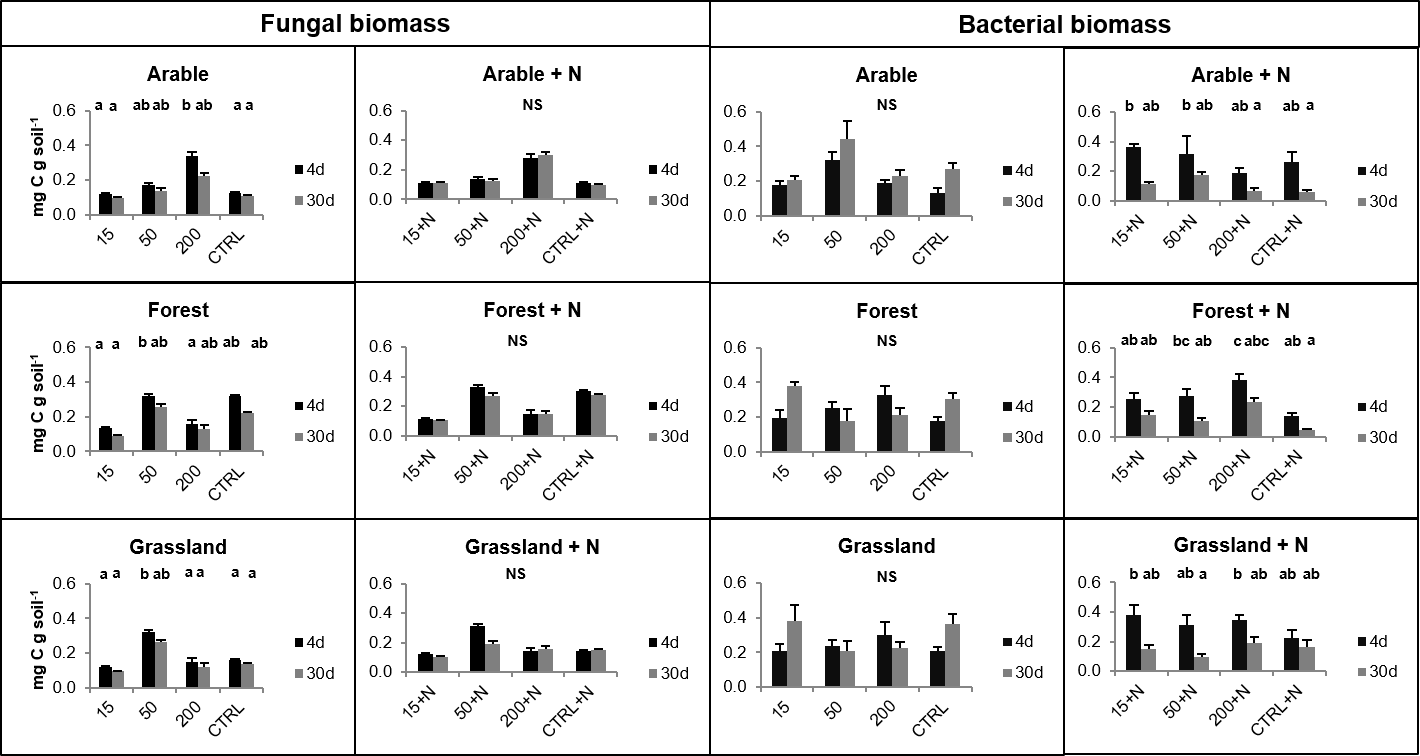

Supplement: S4 Fig — 15, 50 and 200 represent the quantity of C added as equal to 15%, 50%, and 200% of the initial microbial biomass carbon. 4d: fourth day of incubation. 30d: thirtieth day of incubation. CTRL: control treatment. N: NH4NO3. (TIF) [file pone.0216730.s004.tif]
