# Supplementary material for: Effect of the amount of organic trigger compounds, nitrogen and soil microbial biomass on the magnitude of priming of soil organic matter
Source: PLoS One. 2019 May 16;14(5):e0216730. doi: 10.1371/journal.pone.0216730 (PMC6522013; doi:10.1371/journal.pone.0216730)
Supplement: S1 Table — (DOCX) [file pone.0216730.s005.docx]

**Table S1:** Two-way ANOVA results of the effect of amount of glucose additions (15%, 50%, and 200% of the microbial biomass carbon), nitrogen addition (yes/no) and their interactions on the amount of glucose-derived C (µg C-CO_2_ g soil^-1^), primed C (µg C-CO_2_ g soil^-1^). df represents the numerator, denominator degrees of freedom.

| **Soil Type** |  | **Term** | **F** | **df** | **P** |
| --- | --- | --- | --- | --- | --- |
| **Arable** | **Glucose derived C** | Amount of glucose | 32.304 | 2, 18 | < 0.0001 |
|  |  | Nitrogen | 0.249 | 1, 18 | 0.625 |
|  |  | Amount of glucose x nitrogen | 10.653 | 2, 18 | 0.001 |
|  | **Primed C** | Amount of glucose | 3.108 | 2, 18 | 0.074 |
|  |  | Nitrogen | 6.164 | 1, 18 | 0.025 |
|  |  | Amount of glucose x nitrogen | 0.899 | 2, 18 | 0.428 |
| **Forest** | **Glucose derived C** | Amount of glucose | 3.667 | 2, 18 | 0.052 |
|  |  | Nitrogen | 0.231 | 1, 18 | 0.638 |
|  |  | Amount of glucose x nitrogen | 0.645 | 2, 18 | 0.540 |
|  | **Primed C** | Amount of glucose | 0.146 | 2, 18 | 0.865 |
|  |  | Nitrogen | 0.021 | 1, 18 | 0.886 |
|  |  | Amount of glucose x nitrogen | 0.326 | 2, 18 | 0.726 |
| **Grassland** | **Glucose derived C** | Amount of glucose | 8.629 | 2, 18 | 0.003 |
|  |  | Nitrogen | 4.040 | 1, 18 | 0.063 |
|  |  | Amount of glucose x nitrogen | 2.552 | 2, 18 | 0.111 |
|  | **Primed C** | Amount of glucose | 0.367 | 2, 18 | 0.700 |
|  |  | Nitrogen | 1.102 | 1, 18 | 0.312 |
|  |  | Amount of glucose x nitrogen | 1.060 | 2, 18 | 0.373 |
