# Supplementary material for: Effect of the amount of organic trigger compounds, nitrogen and soil microbial biomass on the magnitude of priming of soil organic matter
Source: PLoS One. 2019 May 16;14(5):e0216730. doi: 10.1371/journal.pone.0216730 (PMC6522013; doi:10.1371/journal.pone.0216730)
Supplement: S2 Table — 15, 50 and 200 represent the quantity of C added as equal to 15%, 50%, and 200% of the microbial biomass carbon. N: NH4NO3. *: Significant values (P < 0.05). (DOCX) [file pone.0216730.s006.docx]

**Table S2:** One-sample T test (test value = 0) on significance of primed C-CO_2_ accumulation. 15, 50 and 200 represent the quantity of C added as equal to 15%, 50%, and 200% of the microbial biomass carbon. N: NH_4_NO_3_. *: Significant values (P < 0.05).

| **Treatment** | **t** | **df** | **Sig. (2-tailed)** |
| --- | --- | --- | --- |
|  |  |  |  |
| Arable 15 | 1.632 | 3 | 0.244 |
| Arable 15+N | 10.122 | 3 | 0.002* |
| Arable 50 | 12.038 | 3 | 0.007* |
| Arable 50+N | 3.358 | 3 | 0.078 |
| Ararable 200 | 2.480 | 3 | 0.089 |
| Arable 200+N | 6.220 | 3 | 0.008* |
| Forest 15 | 2.517 | 3 | 0.086 |
| Forest 15+N | 2.803 | 3 | 0.107 |
| Forest 50 | 5.373 | 3 | 0.013* |
| Forest 50+N | 3.196 | 3 | 0.049* |
| Forest 200 | 6.843 | 3 | 0.006* |
| Forest 200+N | 3.458 | 3 | 0.041* |
| Grassland 15 | 1.127 | 3 | 0.377 |
| Grassland 15+N | 8.924 | 3 | 0.003* |
| Grassland 50 | 1.625 | 3 | 0.246 |
| Grassland 50+N | 4.875 | 3 | 0.016* |
| Grassland 200 | 2.620 | 3 | 0.120 |
| Grassland 200+N | 6.974 | 3 | 0.020* |
