# Supplementary material for: Effect of the amount of organic trigger compounds, nitrogen and soil microbial biomass on the magnitude of priming of soil organic matter
Source: PLoS One. 2019 May 16;14(5):e0216730. doi: 10.1371/journal.pone.0216730 (PMC6522013; doi:10.1371/journal.pone.0216730)
Supplement: S3 Table — (DOCX) [file pone.0216730.s007.docx]

**Table S3:** Two-way ANOVA results of the effect of amount of glucose additions (15%, 50%, and 200% of the microbial biomass carbon), nitrogen addition (yes/no) and their interactions on the soil microbial biomass measured at 4^th^ and 30^th^ days of incubation. df represents the numerator, denominator degrees of freedom.

| **Soil type** | **Term** | **F** | **df** | **P** |
| --- | --- | --- | --- | --- |
| **Arable** | Amount of glucose | 6,888 | 7, 48 | < 0.0001 |
|  | Nitrogen | 5,626 | 1, 48 | 0.022 |
|  | Amount of glucose x nitrogen | 4,900 | 7, 48 | < 0.0001 |
| **Forest** | Amount of glucose | 5,096 | 7, 48 | < 0.0001 |
|  | Nitrogen | 3,015 | 1, 48 | 0.089 |
|  | Amount of glucose x nitrogen | 1,531 | 7, 48 | 0.180 |
| **Grassland** | Amount of glucose | 3,909 | 7, 48 | 0.002 |
|  | Nitrogen | 1,600 | 1, 48 | 0.212 |
|  | Amount of glucose x nitrogen | 3,068 | 7, 48 | 0.009 |
